# Body fat and circulating leptin levels in the captive short-beaked echidna (*Tachyglossus aculeatus*)

**DOI:** 10.1007/s00360-024-01559-z

**Published:** 2024-05-15

**Authors:** Kate J. Dutton-Regester, Alice Roser, Haley Meer, Andrew Hill, Michael Pyne, Aiman Al-Najjar, Tim Whaites, Jane C. Fenelon, Katherine L. Buchanan, Tamara Keeley, Marilyn B. Renfree, Stephen D. Johnston

**Affiliations:** 1 School of the Environment, The University of Queensland, Gatton, 4343 Australia; 2 School of Veterinary Science, The University of Queensland, Gatton, 4343 Australia; 3 Currumbin Wildlife Sanctuary, Currumbin, QLD 4223 Australia; 4 Centre for Advanced Imaging, The University of Queensland, Brisbane, 4067 Australia; 5 Queensland X-ray, South Port, QLD 4215 Australia; 6 School of BioSciences, The University of Melbourne, Melbourne, VIC 3010 Australia; 7 School of Life and Environmental Sciences, Deakin University, Geelong, VIC 3216 Australia

**Keywords:** Short-beaked echhidna, Leptin, Adiposity, Monotreme, Body fat

## Abstract

It is possible that the reproductive strategy of the short-beaked echidna is related to seasonal changes in fat deposition and energy availability, regulated by seasonal changes in endocrine function. We predicted that circulating leptin levels would be directly proportional to adiposity during most of the year, but that a change in this relationship would occur during the pre-breeding season to allow increased fat deposition. To test this hypothesis, we made use of a captive colony of echidnas to describe and quantify changes in fat distribution and the adipostatic hormone leptin. First we assessed seasonal changes in circulating leptin levels, body mass and adiposity for three male and three female adult echidnas maintained on a standard diet. Second, we explored the relationship between circulating leptin levels and increased caloric intake for an additional five adult female echidnas that were provided with supplemented nutrition. Third we visualised fat distribution in male and female adult echidnas using magnetic resonance imaging (MRI) before and after the breeding season, to determine where fat is deposited in this species. For echidnas maintained on the standard diet, there were no seasonal changes in body mass, body fat or plasma leptin levels. However, female echidnas provided with supplemented nutrition had significantly elevated plasma leptin levels during the breeding season, compared to the pre-and post- breeding periods. MRI showed substantial subcutaneous fat depots extending dorso-laterally from the base of the skull to the base of the tail, in both sexes. Pre-breeding season, both sexes had considerable fat deposition in the pelvic/rump region, whilst the female echidna accumulated most fat in the abdominal region. This study shows that male and female echidnas accumulate body fat in the pelvic/rump and the abdominal regions, respectively and that circulating leptin may promote fattening in female echidnas during the breeding season by means of leptin resistance. However, further research is required to evaluate the precise relationship between seasonal changes in leptin and adiposity.

## Introduction

In eutherian mammals, the hormone leptin is predominantly synthesised and secreted by adipose tissue and an increase in adipose tissue is concomitant with an increase in circulating leptin concentrations (Zhang et al. 1994; Denver et al. 2011). In addition, leptin can act to directly regulate glucose and lipid metabolism on peripheral tissues such as the pancreas, liver, skeletal muscle and the cardiovascular system (Pereira et al. 2021). Thus, leptin can signal whether adequate energy reserves are available for normal female reproductive function; for example, low circulating leptin concentrations associated with suboptimal energy stores may inhibit reproduction (Denver et al. 2011). Leptin also functions to regulate fat reserves (Friedman-Einat et al. 2014). High fat concentrations result in anorectic behaviour and an increased metabolic rate, resulting in loss of body mass (Denver et al. 2011). However, this mechanism would appear to be counterintuitive for seasonally breeding and hibernating animals that require sufficient seasonal accumulation of energy reserves to support reproduction and/or hibernation. In such animals, while leptin levels are directly proportional to adiposity for most of the year, this relationship changes during the pre-hibernation/reproductive fattening period (Rosseau et al. 2002; Townsend et al. 2008). This change occurs by means of ‘leptin decoupling’, a mechanism whereby leptin levels decrease as body fat increases (Rosseau et al. 2002; Townsend et al. 2008). Alternatively, this change can also result from leptin resistance, in which circulating leptin concentrations correlate with adiposity, but hypothalamic receptors are resistant to the effects of elevated leptin concentrations (Rosseau et al. 2002; Townsend et al. 2008).

Monotremes are an ancient mammalian lineage having diverged from the therian mammal lineage about 166 million years ago (Warren et al. 2008; Luo et al. 2011). While monotremes lay eggs, they also possess a yolk sac placenta *in utero* for the transport of nutrients during the early stages of foetal development in a similar manner to viviparous therian mammals (Griffiths 1978). Monotremes, therefore, represent an intermediate stage between oviparous reptiles and viviparous therian mammals. While leptin is a hormone that is conserved across vertebrate groups (Denver et al. 2011), its role as an adipostatic factor remains inconclusive in reptiles (Paolucci et al. 2001; Spanovich et al. 2006) and there are conflicting reports across avian species (Kuo et al. 2005; Cerasale et al. 2011; Gogga et al. 2013; Churchman and MacDougall-Shackleton 2022; Rossi et al. 2023). Consequently, there is speculation that the adipostatic function of leptin may have been acquired during the evolution of mammals (Sprent et al. 2012; Michel et al. 2016). However, to the authors knowledge, marsupials are yet to be investigated. While Sprent et al. (2012) reported a weak negative relationship between circulating leptin levels and body mass in the short-beaked echidna, a monontreme, body mass may not accurately reflect body fat levels (Spady et al. 2009; Garcia et al. 2011; Zhao et al. 2014). Therefore, further studies that measure adiposity directly are important to help clarify whether leptin plays a role in signalling adiposity in this taxa.

The short-beaked echidna is a seasonally reproducing species. Reproduction can be particularly demanding for female echidnas, making this species an excellent model for studying the mechanistic links between nutritional supply, leptin and body composition. In wild populations, echidnas only reproduce once every 2–6 years (Rismiller and McKelvey 2000; Morrow et al. 2009). However, under captive husbandry, echidnas have been recorded to reproduce in consecutive years (Ferguson and Turner 2013; Wallage et al. 2015; Dutton-Regester et al. 2021). This contrast between wild and captive reproductive timings is possibly due to the provision of increased nutritional supply to female echidnas in captivity before and during the breeding season (allowing for fat deposition and maintenance) and the provision of burrow boxes that have been suggested, but not proven, to facilitate energy conservation associated with thermoregulation (Wallage et al. 2015). The literature from wild echidnas suggests a circannual pattern of body mass, with body mass increases post breeding season concomitant with increased foraging behaviour, and that body mass reaches its maximum just before the hibernation or the breeding season (Smith et al. 1989; Abensperg-Traun and Boer 1991; Morrow et al. 2016; Nicol and Anderson 2007). Based on these observations, it is possible that reaching a sufficient or threshold fat deposition could influence breeding success.

Here, we sought to assess the relationship between body composition, nutritional supply and circulating leptin concentrations in a successful captive breeding colony of short-beaked echidnas housed at Currumbin Wildlife Sanctuary (CWS). Our objectives were to (1) investigate the gene expression of leptin and its receptor, (2) assess seasonal changes in adiposity and circulating leptin levels, (3) explore the relationship between circulating leptin levels and increased caloric intake, and (4) visualise fat distribution in both male and female adult echidnas using magnetic resonance imaging (MRI) before and after the breeding season, to determine where fat is deposited in this species. We predict that circulating leptin levels would be directly proportional to adiposity during most of the year, but that a change in this relationship would occur during the pre-breeding season to allow increased fat deposition.

## Materials and methods

### Animals and husbandry

This study was conducted between 2018 and 2021 and included eight female and three male, sexually mature echidnas (*Tachyglossus aculeatus aculeatus)* from the captive population at CWS (28.1356° S, 153.4886° E, Gold Coast, Australia; Table 1). All echidnas had previously reproduced except for F3. Female echidnas were housed, singly or in pairs, in a specialised echidna breeding centre consisting of ten enclosures (4.8 m × 3.8 m; Wallage et al. 2015). This study was approved by the University of Queensland Animal Ethics Committee (SAFS/317/20).

**Table 1:** Weight, age and the study in which each echidna participated

Echidna ID	Average weight during study period (kg)	Age of echidna (years)	Study echidna was involved in	Received supplemented diet May–Oct (Y/*N*)	Involved in reproductive studies (Y/*N*)
F1^a^	4.2	> 22^a^	MRI & Leptin	Y	Y
F2	4.5	6	DEXA & Leptin	N	N
F3	3.8	9	DEXA & Leptin	N	N
F4	3.7	9	DEXA & Leptin	N	N
F5^a^	4.1	> 22^a^	Leptin	Y	Y
F6^a^	3.8	> 22^a^	Leptin	Y	Y
F7^a^	4.3	> 22^a^	Leptin	Y	Y
F8^a^	4.7	> 22^a^	Leptin	Y	Y
M1	4.6	+ 21^a^	MRI & DEXA & Leptin	N	Y
M2	4.6	+ 20^a^	DEXA & Leptin	N	Y
M3	5.5	+ 20^a^	DEXA & Leptin	N	Y

^a^ Wild-caught animals—precise ages unknown

In the non-breeding season, male echidnas were group housed in an off-exhibit enclosure (6.0 m × 3.0 m) but during the breeding season (July–October) they were periodically paired with females (F1, F5–F8) as part of other studies (See Dutton-Regester et al. 2021; Dutton-Regester et al. 2022). All enclosures were filled with a combination of sand and leaf litter substrate. Each enclosure contained logs, rocks, browse and enrichment items (e.g. termite mounds and toys). Female enclosures also had one or two burrow boxes and a heat lamp that was switched on at 15:00 h and off on the following morning at 10:00 h to provide a heat source during the cool of the night. All echidnas were provided with 100 g daily of a beef mince—based maintenance diet (Table 2). As echidnas were not always housed singly, it was not possible to monitor individual food consumption or calorific intake. However, the captive echidnas used in this study generally consume all food provided, with the exception of the final 24 h before gestation (Dutton-Regester et al. 2022). Between the beginning of May through to the end of October each year, the diet of F1 and F5–F8 was supplemented with additional fly pupae and olive oil and increased from 100 to 150 g per day as these females were part of a breeding program in which they were paired with males from July; eggs and pouch young that were produced were collected at various stages of development for a parallel study (See Dutton-Regester et al. 2021; Dutton-Regester et al. 2022); male echidnas and females F2–F4 did not receive this extra ration.

**Table 2:** Nutritional information for maintenance and reproductive echidna diets used during the study

	Recipe quantity	Calories/100 g	Calories/recipe quantity	Fat (g)/100 g	Fat (g)/recipe quantity
Maintenance diet					
Premium beef mince (coles)	750 g	137	1027.5	5	37.5
3 eggs (coles)	177 g	143	253.1	10	17.7
Fly pupae (pisces enterprises)	25 g	616.6	154.2	11.3	2.8
Wheat bran (coles)	60 g	268	160.8	4.1	2.5
Olive oil (coles)	50 ml	884	419	100	47.4
Dextrose monohydrate (independent own)	160 g	372	595.2	0	0
Balanced cal (mavlab)	5 g	28	1.4	0	0
Soluvite D (vetafarm)	12.5 g	180	22.5	0	0
Vitamin E (white E)	20 g	Negligible	Negligible	0	0
*Calories and fat/100 g portion fed to echidna*			*212.9*		*8.7*
Reproductive diet					
Premium beef mince (coles)	750 g	137	1027.5	5	37.5
Egg (coles)	177 g	143	253.1	10	17.7
Fly pupae (pisces enterprises)	45 g	616.6	277.5	11.3	5.1
Wheat bran (coles)	60 g	268	160.8	4.1	2.5
Olive oil (coles)	100 ml	884	838	100	94.8
Dextrose monohydrate (independent own)	160 g	372	595.2	0	0
Balanced cal (mavlab)	5 g	28	1.4	0	0
Soluvite D (vetafarm)	12.5 g	180	22.5	0	0
Vitamin E (white E)	20 g	Negligible	Negligible	0	0
*Calories and fat/150 g portion fed to echidna*			*365.3*		*18.1*

General behavioural records (e.g. eating, torpor, activity levels) for females F2, F3 and F4 were provided by CWS zookeepers. Behavioural data for females F1, F5–F8 and all male echidnas were collected as part of other studies (Dutton-Regester et al. 2021; Dutton-Regester et al. 2022) during which their behaviour was monitored daily between July to October; behaviour records were otherwise unavailable. Daily temperature and rainfall were accessed from the Bureau of Meteorology (Coolangatta Bureau Station, no. 40,717, 28.17° S, 153.51° E, Gold Coast, Australia, http://www.bom.gov.au/climate/).

### Dual-energy X-Ray absorptiometry (DEXA)

To quantify total body fat percentage, echidnas were transported to Queensland X-Ray (coordinates 27.97265, 153.40946, Gold Coast, Australia) for DEXA scans. The DEXA (Medilink, Medix DR, Australia) procedure was initially validated against Computerised Tomography (CT; Siemens, Somatom Perspective 128, 130Kv, Germany) using three echidna cadavers. These cadavers were opportunistically sourced from roadkill, from the Toowoomba and Gatton regions of south-east Queensland. Cadavers were frozen and stored at The University of Queensland, Gatton campus (27.5512° S, 152.3355° E, Gatton, Australia) until required for CT scanning. For CT scanning, these echidnas were thawed and placed in a prone position for the 20 s scan. Total body and fat volumes were calculated using the ‘volume measurement tool’ available on the CT scanner. This volume calculation method utilises the Hounsfield Unit (HU) value of tissues, which describes how tissues attenuate/absorb x-rays in CT scans. The software can display the volume of a user selected HU range. Therefore, the HU range can be set to include all tissues, soft and bone, but also a limited range to only show fat density tissues. A ratio can then be calculated from the two volume calculations. The ranges used for fat only and total body volumes were −150 to −20HU and −150 to 2000HU, respectively. CT scanning parameters included dose modulation, 200; detector configuration, 32 × 0.6 mm; rotation time, 0.6s; pitch, 0.5. For DEXA scanning, each echidna was anaesthetised using isoflurane as described for the MRI scans and placed in ventral recumbency on the DEXA platform. While being closely monitored by a veterinarian, the echidnas remained under anaesthesia for the duration of the 45 s DEXA scan. Scanning parameters were whole body at a speed of 280 mm/sec and a resolution of 2 mm. Analytic zones were modified to only include thorax, abdomen and pelvic regions. The thorax region was modified to include the head and arms. The pelvic region was adjusted to include the leg (distal from the origin of the leg soft tissues) and tail; thereby accounting for a whole-body coverage. The abdominal zone automatically adapts between the aforementioned regions and covers part of the abdomen and pelvis. The distal limb regions were positioned extended away from the echidna’s body and not analysed. After validation, echidnas F2–F4 and M1–M3 were transferred to Queensland x-ray every three months from October 2018 to October 2019 (5 scans total).

### Leptin and leptin receptor gene expression

Adult male and female echidna fat and other tissues were collected opportunistically from injured animals brought into Currumbin Wildlife Hospital (28.1356° S, 153.4886° E, Gold Coast, Australia) that required euthanasia for animal welfare reasons. Tissues collected were either stored in RNAlater (Thermo Fischer Scientific, Waltham, MA, USA) or snap frozen in liquid nitrogen. Total RNA was extracted from the fat tissues with the RNeasy Lipid Tissue Mini kit (Qiagen, Germany) and from all other tissues with the GenElute Mammalian total RNA Miniprep Kit (Sigma-Aldrich, St Louis, MO, USA). All RNA was then DNase treated using DNA-free (Ambion, Thermo Fischer Scientific, Waltham, MA, USA) according to the manufacturer’s instructions. The quality and quantity of RNA was verified by optical density reading using a NanoDrop ND-1000 spectrophotometer (BioLab, Thermo Fisher Scientific, Waltham, MA, USA). One microgram total RNA was then reversed transcribed using the Superscript IV kit (Invitrogen, Thermo Fischer Scientific, Waltham, MA, USA) with oligo(dT) priming according to the manufacturer’s instructions. RT-PCR was carried out using echidna-specific primers for *GAPDH* as the reference gene, *LEPTIN* and *LEPTIN RECEPTOR* (*LEPR*, Table 3) using the GoTaq Green Master Mix (Promega, Wisconsin, USA) as per manufacturer’s instructions for the 25 uL reaction volume with 0.2 µM of forward and reverse primers and 0.5 µL of sample cDNA template with Nuclease-free H_2_O instead of template used as the negative control. PCR reactions were run on a MJ Research PTC-100 (Bio-Rad, California, USA) and amplified with an initial denaturation at 94 °C for 5 min, followed by 40 cycles of denaturation at 94 °C for 30 s, annealing at 60 °C for 30 s and extension at 72 °C for 30 s, followed by a final extension at 72 °C for 5 min. Amplification products were observed by electrophoresis on a 1% agarose gel at 100 V for 50 min, followed by visualization under UV with a ChemiDoc XRS + Imaging System (Bio-Rad, California, USA).

**Table 3:** Primers used for RT-PCR

Product	Forward 5’-3’	Reverse 5’-3’
*GAPDH*	CAGAAGACGGTAGATGGCCC	TCAGATCCACCACACGGTTG
*LEPTIN*	ATCCAGGCTGACACCAAAAC	GCATAGGCAGACTGGAGAGG
*LEPR*	GTGGACCCAAAGACATTGCT	TCCCGACAAGAGGTAGATGG

### Venipuncture, blood collection and body mass

Monthly blood samples were collected between 10:00–12:00 from echidnas housed at CWS between July 2018 to October 2019 and May 2018 to October 2019 for echidnas M1–M3, F2–MF and F1, F5–F8, respectively. While the echidnas were under isoflurane anaesthesia (Abbott’s Australasia PtyLtd), a blood sample (approximately 2 mL) was recovered from the rostral sinus as previously described by Johnston et al. (2006) using a 25 g butterfly needle (Terumo, Surflo, Japan) and 3 mL syringe (Zebravet, China). The blood sample was placed in a heparinised collection tube (Sarstedt, Germany) and then centrifuged at 1000 g for 10 min. Plasma was collected and stored at −20 °C until analysis. For the months that involved DEXA scans, blood samples were collected 6–7 days before conducting the scan as the required resources (e.g. procedure bench, centrifuge, sample storage) were not available onsite. To investigate the accuracy of using mass percentage (Sprent et al. 2012) as an indirect measure of adiposity, monthly weights for the periods January 2017–July 2018 and November–December 2019 were obtained for echidnas M1–M3 and F2–F4 from CWS zookeeper records (resulting in three years of monthly weight data per echidna) and added to the monthly weight records obtained while under isoflurane anaesthesia (July 2018–October 2019). A mean weight was calculated for each echidna, and the weight measured at the time of blood sampling was converted to a percentage of the mean for that animal (Sprent et al. 2012).

### Plasma leptin analysis

Plasma samples (100 µL in duplicate) were analysed for leptin concentration using a multi-species leptin radioimmunoassay (RIA) as per the manufacturer’s recommended procedures (Leptin RIA, Catalogue XL-85 K, Merck, Victoria, Australia) and as previously validated for the short-beaked echidna by Sprent et al. (2012). The limit of detection (LOD) for this assay was 1 ng/mL (Human Equivalent: HE). Counts per minute (CPM) were used to evaluate CV% with values under 12% considered acceptable. The control values were within the reported range (mid to upper 10% of the range) as specified by the manufacturer. CPM counts between replicates were averaged and then the average NSB (non-specific binding) was subtracted from all values (zeros, standards and samples). The percent binding was then calculated as the average CPM for each standard or unknown, divided by the average CPM for the zero standard. Percent binding values of standards, controls and samples were used for the data extrapolation process. Standard concentrations were log transformed and plasma sample leptin values were extrapolated using a non-linear regression (curve fit) using a sigmodal dose-response (variable slope), weighed by 1/Y^2^ with a least squares regression in GraphPad Prism 9.1. A weighted 4-parameter analysis was also tested (as recommended by the manufacturer) which calculated similar results. Samples which had inadequate volumes (< 200 ≥ 100 µL) were analysed without duplication (e.g. single replicate of 100 µL; *n* = 13 samples) or analysed without duplication with a volume less than recommended (e.g. 80 µL with volume made up to 100 µL with assay buffer; *n* = 3 samples). Those samples with less than 100 µL had their extrapolated leptin concentration values adjusted for volume after extrapolation. Samples with concentrations below the limit of detection (LOD; M1: *n* = 3, F1: *n* = 2, F5: *n* = 6, F6: *n* = 9, F7: *n* = 4) were substituted with a constant value of 0.5 ng/mL (i.e. half the assay’s LOD). All values were expressed in ng/mL (HE).

### Magnetic resonance imaging (MRI) scans

To observe and topographically localise the distribution of adipose tissue, echidnas F1 and M1 were transferred to the Centre for Advanced Imaging at the University of Queensland, St Lucia campus (27.4975° S, 153.0137° E, Brisbane, Australia), once during December 2020 and once during June 2021 for MRI scans. Echidnas were imaged on a 3 Tesla Prisma^fit^ (Siemens, Erlangen, Germany) system using a 32 Channel coil and 18 Body matrix coil. The parameters of T2-weighted spin-echo images were TR, 3350ms; TE, 101ms; FoV, 450 mm; slice thickness, 3 mm; and number of slices, 30. The parameters of T1-weighted spin-echo images were TR, 7.0ms; TE, 2.52ms; FoV, 300 mm; slice thickness, 1 mm; and number of slices, 280/slab. One at a time, each echidna was placed on its ventral surface on the MRI bed. To ensure that echidnas would remain stationary during the scanning procedure, they were placed under isoflurane anaesthesia; the anaesthetic was maintained with 1% isoflurane (Abbott’s Australasia PtyLtd) delivered at 1.5 L of oxygen per minute and administered by a mask, and closely monitored by a veterinarian until completion of the procedure (approximately 30 min). Magnetic resonance images were exported as DICOM format files which were subsequently viewed and annotated using OSIRIX DICOM (http://homepage.mac.com/rossentantoine/osirix/) viewer software on an Apple MacBook Air M1 computer. A male and female coronal slice (3 mm) DICOM file from a standardised location on the echidna was then transferred to MedSeg, an online segmentation tool (https://htmlsegmentation.s3.eu-north), to quantify the volume (mL) of adipose tissue before and after the breeding season. Adipose tissue segmentation was performed by carefully ‘painting’ visible tissue during which the segmentation software isolates the individual volumes of fat using a k-means clustering algorithm. The volume (mL) of adipose tissue was determined for individual regions (cranial, thoracic, abdominal, pelvic/rump) and the whole body. Each segmentation was performed three times by a single observer to assess intra-observer reliability, in which a value of ≤ 0.20, was regarded as poor, 0.21–0.40 as fair, 0.41–0.60 as moderate, 0.61–0.80 as good and 0.81–1.00 as very good agreement (McHugh 2012).

### Statistical analysis

Non-parametric statistical tests were used throughout, due to the relatively small sample sizes available for comparison. The relative (or percent) change in body fat between December to June MRI scans was calculated as (final value − initial value/initial value) × 100%. A Pearson correlation coefficient was used to investigate the relationship between CT and DEXA scans. For the purposes of data analysis, body fat percentage, body mass and leptin data were classified into three periods; pre-breeding season (April–June), breeding season (July–October) and post-breeding season (November–February). These data were analysed separately for males (which consumed a standard diet throughout study period and were involved in reproductive activity), females F2–F4 (which consumed a standard diet throughout study period but were not involved in reproductive activity) and females F1 and F5–F8 (which were provided with a supplemented diet May–Oct and were involved in reproductive activity between July to October). As male and female echidnas can differ significantly in physiological condition between years (Nicol and Morrow 2012; Sprent et al. 2012), individual echidnas from different years were treated as though they were statistically independent. Variations in body fat perentage, body mass and plasma leptin levels were investigated using a Kruskal Wallis ANOVA. Significant differences between groups were explored using Dunn’s multiple comparisons test. Relationships between body fat percentage, mass percentage, body mass and plasma leptin levels were investigated using a Spearman’s rank correlation coefficient as were the relationships between plasma leptin with temperature (on the day of sample collection) and rainfall (on the day of sample collection). For echidnas involved in studies during the breeding season (M1–M3, F1 and F5–F8), the relationship between plasma leptin levels and activity was assessed using rank point biserial correlation in which 0 = male/female engaged in reproductive activity at the time of sample collection (i.e. training, copulatory attempts), 1 = male/female not engaged in reproductive activity at the time of sample collection. Correlations were interpreted using the guidelines of Landis and Koch (1977) in which: 0–0.10 = negligible, 0.11–0.39 = weak, 0.40–0.69 = moderate, 0.70–0.89 = strong, 0.90–1.00 = very strong. Statistics were performed using GraphPad Prism 9.1. All data are presented as means ± S.D., unless indicated otherwise.

## Results

### Leptin and leptin receptor gene expression

**Fig. 1:**
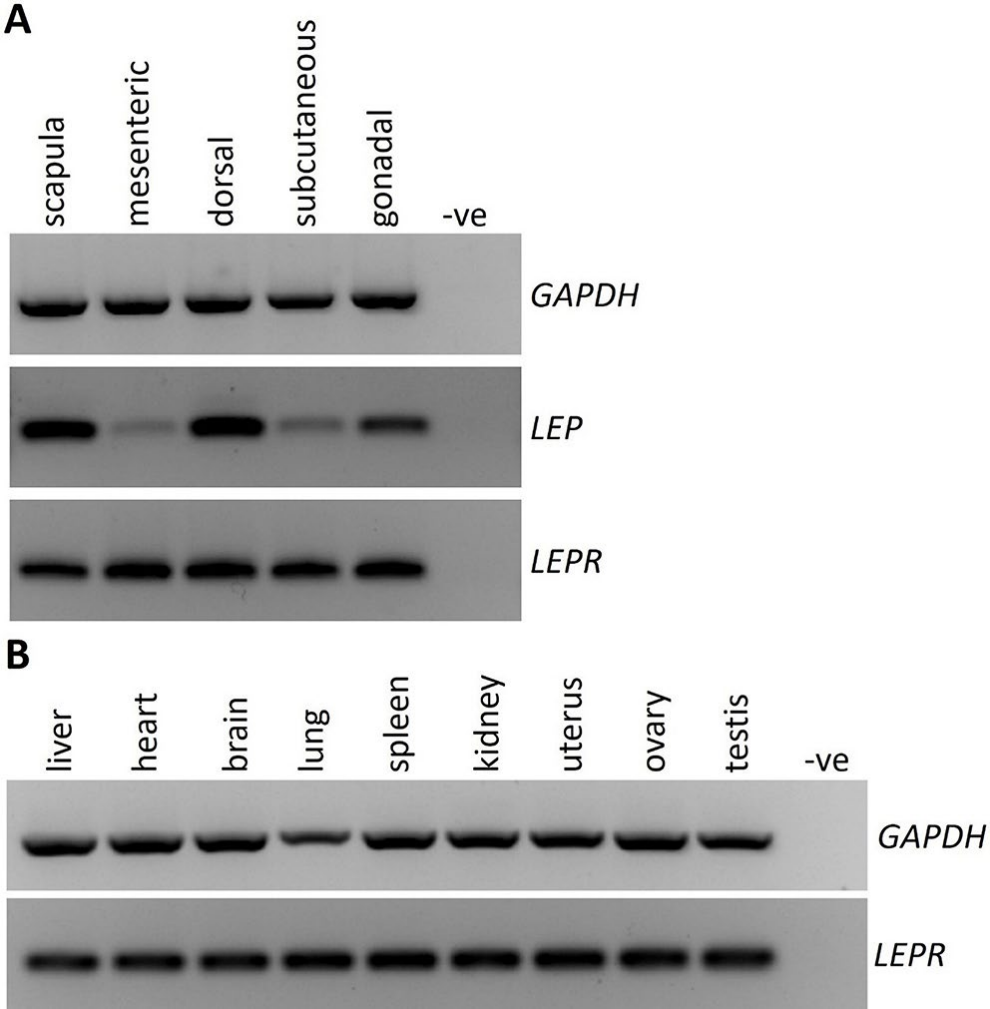
*LEPTIN* and *LEPTIN RECEPTOR* (*LEPR*) RT-PCR gene expression profiles. **A** Both *LEP* and *LEPR* were present in all fat tissues collected from different areas of the body; **B***LEPR* was present in all adult organs examined in both males and females. *GAPDH*: reference gene

RT-PCR was used to assess the expression of *LEPTIN* and the *LEPTIN receptor (LEPR)* across a range of fat tissues (scapula, mesenteric, body, subcutaneous and gonadal). Both *LEPTIN* and *LEPR* were expressed in all fat tissues examined (Fig. 1A). In addition, LEPR was present across a range of organs in the body, including the uterus, ovary and testis (Fig. 1B).

### DEXA validation of body fat

The volume of adipose tissue was removed from the total volume of echidna body tissue, resulting in high correlation between CT and DEXA scans (*r* = 0.95; *p* < 0.05).

### Seasonal changes in body mass, body fat and plasma leptin

#### Body mass

There was no statistically significant change in body mass between periods for males (*p* > 0.05), females maintained on the standard diet (F2–F4; *p* > 0.05) or females provided with additional nutrition (F1 and F5–F8; *p* > 0.05; Table 4). During the breeding (5.0 ± 0.48 kg) and post-breeding (4.8 ± 0.61 kg) seasons, male echidnas had significantly higher body mass than females (F2–F4) maintained on the standard diet (breeding season: 4.1 ± 0.60 kg, *p* < 0.01; post breeding season: 4.1 ± 0.45 kg, *p* < 0.01; Table 4) and as well as females (F1 and F5–F8) provided with supplemented nutrition (breeding season: 4.2 ± 0.42 kg, *p* < 0.05; post breeding season: 4.1 ± 0.34 kg, *p* < 0.05; Table 4).

**Table 4:** Mean body mass (kg), body fat (%) and plasma leptin concentrations for male and female echidnas during the pre-breeding season (April–June), breeding season (July–October) and post-breeding season (November–February) periods

Period	Body mass(kg) Mean ± S.D	Range	Body fat (%) Mean ± S.D	Range	Leptin (ng/mL) Mean ± S.D	Range
Males						
Pre-breeding season	5.0 ± 0.61	4.4, 5.8	24.8 ± 4.99	21.8, 30.6	6.4 ± 6.12	0.5,16.8
Breeding season	5.0 ± 0.48	4.3, 6.1	23.2 ± 4.02	19.0, 28.4	8.9 ± 8.05	0.5, 30.2
Post-breeding season	4.8 ± 0.61	4.1, 5.7	23.3 ± 2.63	21.3, 26.3	10.8 ± 7.62	1.3, 21.5
Females—standard diet						
Pre-breeding season	4.3 ± 0.39	3.7, 5.0	31.3 ± 4.86	27.6, 36.8	6.6 ± 2.81	2.9, 12.1
Breeding season	4.1 ± 0.60	3.7, 4.8	30.1 ± 5.32	21.8, 35.9	8.6 ± 4.00	3.0, 18.7
Post-breeding season	4.1 ± 0.45	3.5, 4.4	29.9 ± 5.03	26.9, 35.7	9.4 ± 2.50	5.9, 13.8
Females—supplemented diet						
Pre-Breeding season	4.4 ± 0.35	3.7, 5.1	–	–	3.7 ± 4.49	0.5, 16.0
Breeding season	4.2 ± 0.42	3.6, 5.1	–	–	5.4 ± 3.78*	0.5, 15.3
Post-breeding season	4.1 ± 0.34	3.6, 4.8	–	–	2.2 ± 1.77	0.5, 5.7

*S.D.* standard deviation

* Statistically significant to both the pre- and post-breeding seasons

### Body fat

There was no statistically significant change in body fat% between periods for males (*p* > 0.05) or females maintained on the standard diet (F2–F4; *p* > 0.05, Table 4). Female echidnas had significantly higher body fat% than males during the pre-breeding season (female: 31.3 ± 4.86% vs. male: 24.8 ± 4.99%; *p* < 0.05) but not in the post-breeding season (female: 29.9 ± 5.03 vs. male: 23.3 ± 2.63; *p* > 0.05); there were inadequate data available to compare body fat percentage during the breeding season, but overall, female echidnas had significantly higher body fat than males (30.4 ± 0.79 vs. 23.8 ± 0.89; *p* < 0.01).

### Plasma leptin concentrations

**Fig. 2:**
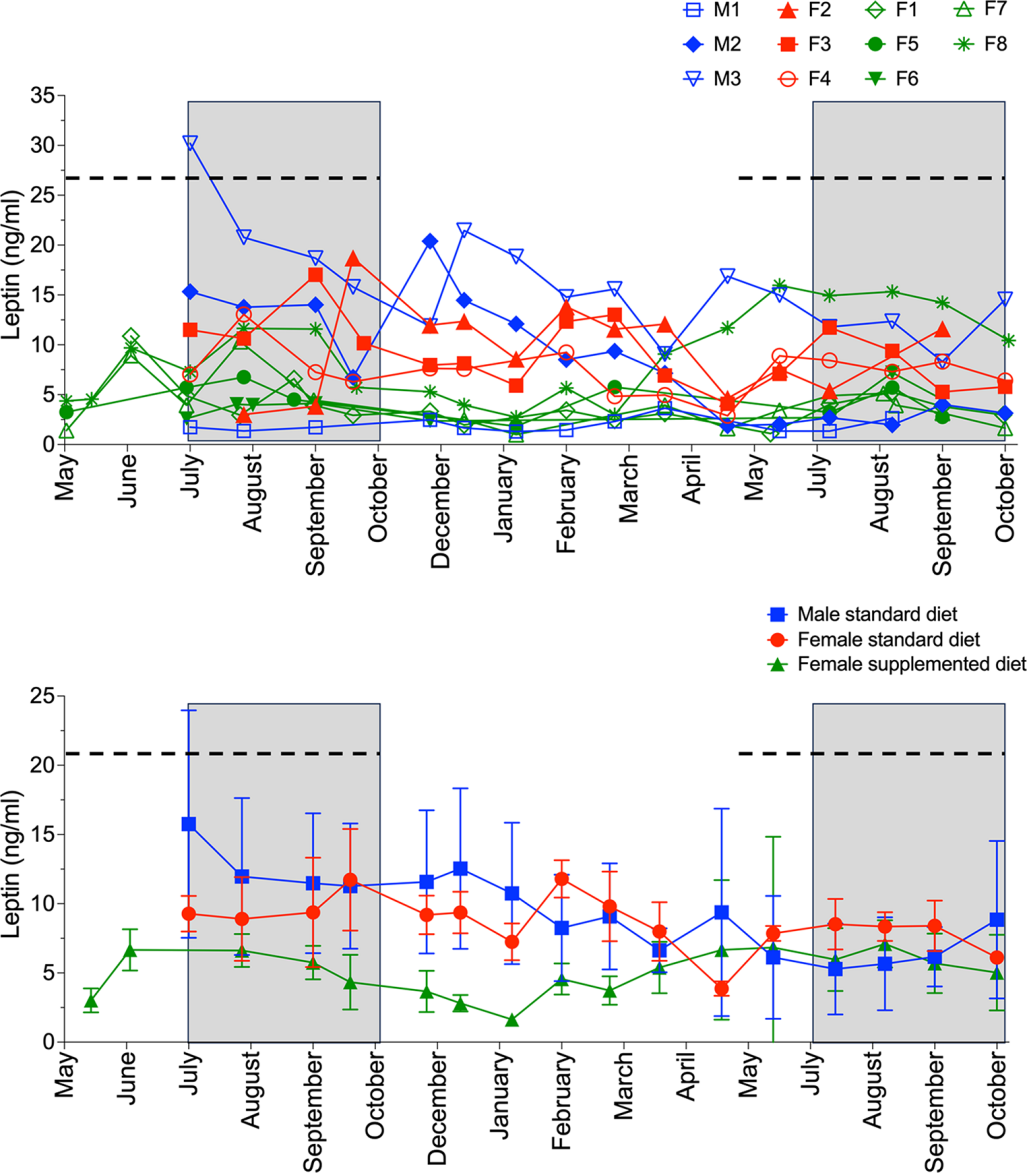
**a** Plasma leptin concentrations (ng/mL) of three male (blue) and three female (red) echidnas maintained on a standard diet, and five female echidnas provided with supplemented nutrition (green) from May to October (indicated by the dotted line). Grey shading indicates the breeding season (July–October). **b** Mean plasma leptin concentrations (ng/mL) of three male (blue) and three female (red) echidnas maintained on a standard diet, and five female echidnas provided with supplemented nutrition (green) from May to October (indicated by the dotted line). Grey shading indicates the breeding season (July–October)

There was no statistically significant change in plasma leptin concentrations between periods for males (*p* > 0.05) or females maintained on the standard diet (F2–F4; *p* > 0.05; Table 4). Females provided with additional nutrition (F1 and F5–F8) had significantly higher plasma leptin levels during the breeding season (5.4 ± 3.78 ng/mL) compared to the pre-breeding (3.7 ± 4.49 ng/ mL; *p* < 0.05) and post-breeding seasons (2.2 ± 1.77 ng/mL; *p* < 0.05). During the post breeding season, plasma leptin levels were significantly higher in males (10.8 ± 7.62 ng/mL; *p* < 0.01) and females maintained on the standard diet (F2–F4; 9.4 ± 2.50 ng/mL; *p* < 0.01) compared to females provided with supplemented nutrition (F1 and F5–F8; 2.2 ± 1.77 ng/mL). Plasma leptin concentrations were highly variable within and between individual echidnas (Fig. 2a, b).

### Relationships between body mass, body fat and plasma leptin

For male echidnas, while there was no significant correlation of body fat percentage with body mass (rs: −0.38; *p* > 0.05) or plasma leptin levels (rs: −0.17; *p* > 0.05), there was a significant, strong negative correlation of body mass with leptin (rs = −0.70, *p* = < 0.05; Table 5). For female echidnas maintained on the standard diet (F2–F4), there was no significant correlation of body fat percentage and body mass (rs = 0.52, *p* > 0.05), body fat percentage and plasma leptin levels (rs = −0.15, *p* > 0.05), or body mass and plasma leptin levels (rs = −0.53, *p* > 0.05; Table 5). For female echidnas provided with a supplemented diet (F1 and F5–F8), body mass was not correlated with leptin (rs = 0.02, *p* > 0.05).

**Table 5:** Spearman rank correlations between body fat, body mass and leptin concentrations in male and female short-beaked echidnas

Variables	Correlation (*p* value)		
	Male	Female—standard diet	Female—supplemented diet
Body fat (%) and body mass (kg)	−0.38 (> 0.05)	0.52 (> 0.05)	–
Body fat (%) and leptin (ng/ml)	−0.17 (> 0.05)	−0.15 (> 0.05)	–
Leptin (ng/ml) and body mass (kg)	−0.70 (< 0.05) *	−0.53 (> 0.05)	0.02 (> 0.05)
Body fat (%) and mass%	−0.23 (> 0.05)	0.54 (> 0.05)	–
Body mass (kg) mass%	0.12 (> 0.05)	0.17 (> 0.05)	

* Statistically significant

### Relationship between body fat percentage, weight, and mass percentage

For male and female echidnas maintained on the standard diet, there was no significant correlation of mass percentage with body fat percentage (males: rs = −0.23, *p* > 0.05; females: rs = 0.54, *p* > 0.05) or body mass (males: rs = 0.12, *p* > 0.05; females: rs = 0.17, *p* > 0.05; Table 5).

### Relationships between leptin and ambient temperature and behaviour

**Fig. 3:**
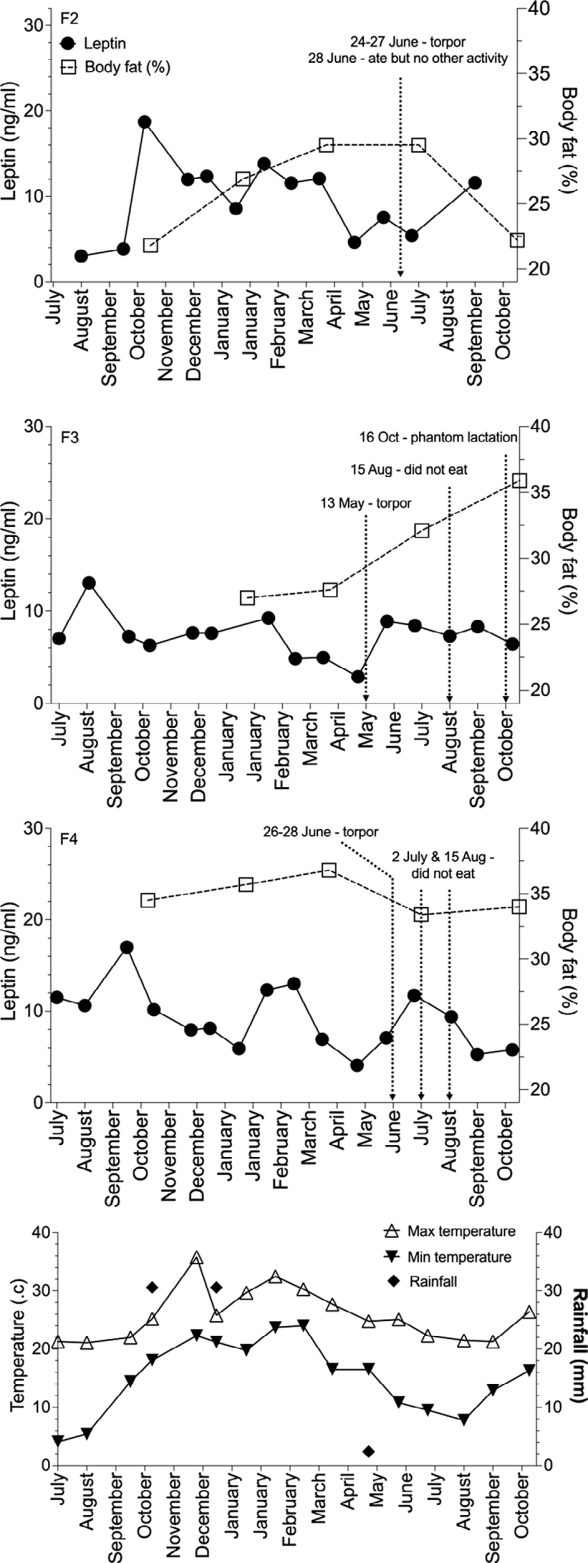
Female echidna body fat percentage and plasma leptin concentrations mapped against changes in temperature and rainfall during the 18-month study

**Fig. 4:**
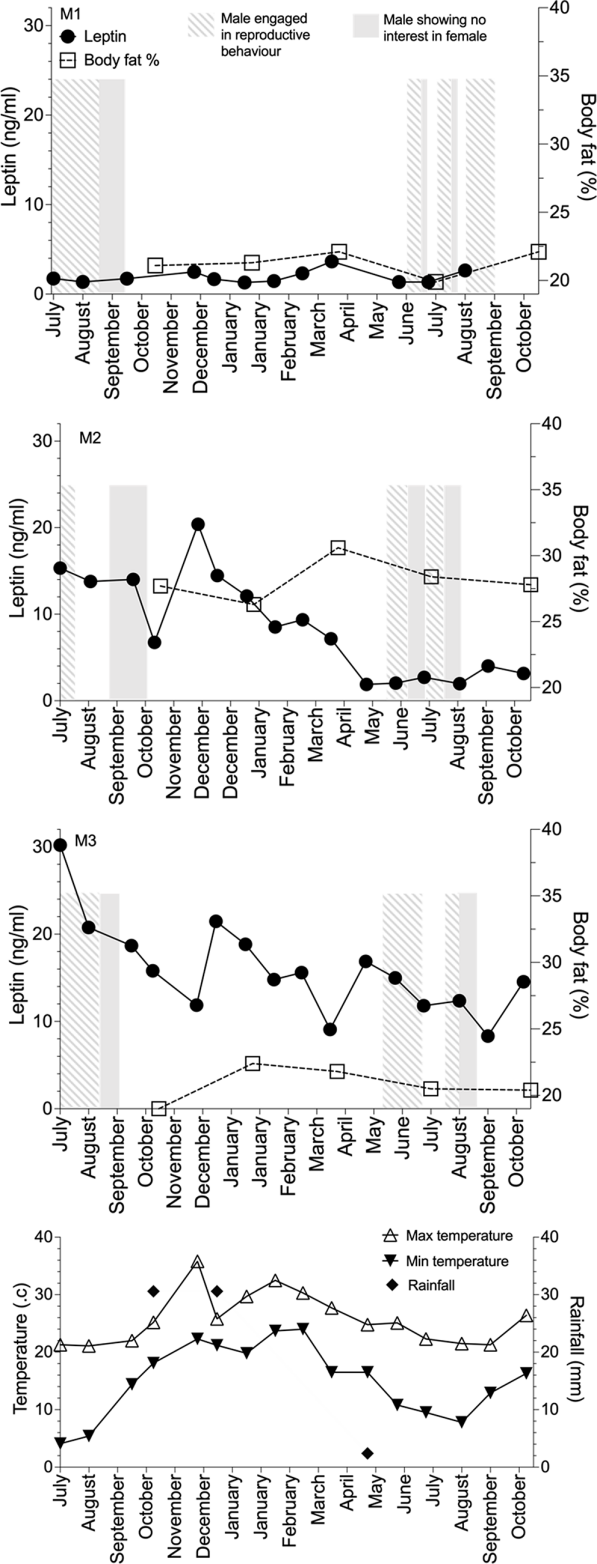
Male echidna body fat percentage and plasma leptin concentrations mapped against changes in temperature and rainfall during the 18-month study

For echidnas M1–M3 and F2–F4, there was no significant correlation of leptin with ambient temperature or rainfall (all *p* values > 0.05; Figs. 3 and 4). Females F2–F4 entered torpor (a phase of reduced metabolism and body temperature, typically restricted to the animals’ resting phase during the day) once in 2019 when minimum daily temperature fell below 18 and 15 °C in May (F3) and June (F2 and F4), respectively (Fig. 3). However, this was only for a maximum of four consecutive days. Leptin concentrations were near or at their lowest levels during a similar period for females F2–F4 (F2: May–July, F3: March–May, F4: May); females F2 and F3, were in torpor during their respective low leptin period, but F4 was in torpor as leptin concentration increased. F4 did not eat on 15th August 2019 and although a blood sample was collected the next morning, there was no notable effect on leptin concentration. While food consumption was not recorded for each individual animal, typically, when individually housed, each animal ate their designated ration to completion, but when two females were housed together or a female was paired with a male it was not possible to ascribe a calorific value consumed to each animal. When male (M1–M3) and female (F1 and F5–F8) echidnas were paired for the breeding season (intermittently between June – October), regardless of whether echidnas were actively engaged in reproductive behaviour (e.g. training, copulatory attempts) or showing no interest in each other, there was no significant difference in leptin concentrations (males: r_rb_ = 0.23, *p* 0.45, Fig. 4; females r_rb_ = 0.36, *p* 0.58).

### Distribution of adipose tissue

**Fig. 5:**
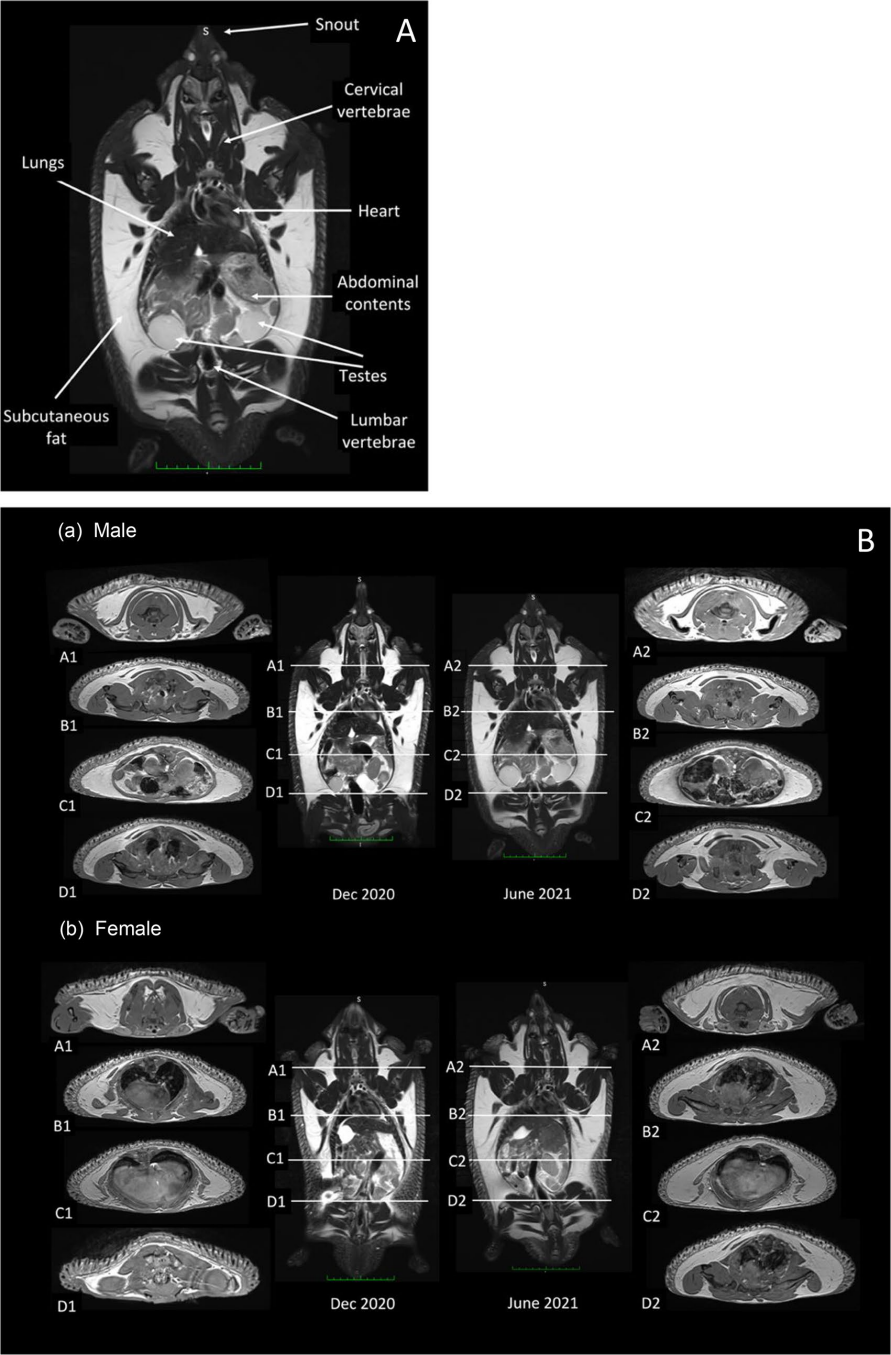
**A** Labelled coronal MRI scans showing fat distribution and organ location; **B** Coronal MRI scans with transverse slices demonstrating fat distribution in a male (*a*) and female (*b*) echidna two months after the end of (December 2020) and one month before (June 2021) the echidna breeding season (July–October). Green scale bar—1 cm divisions

**Fig. 6:**
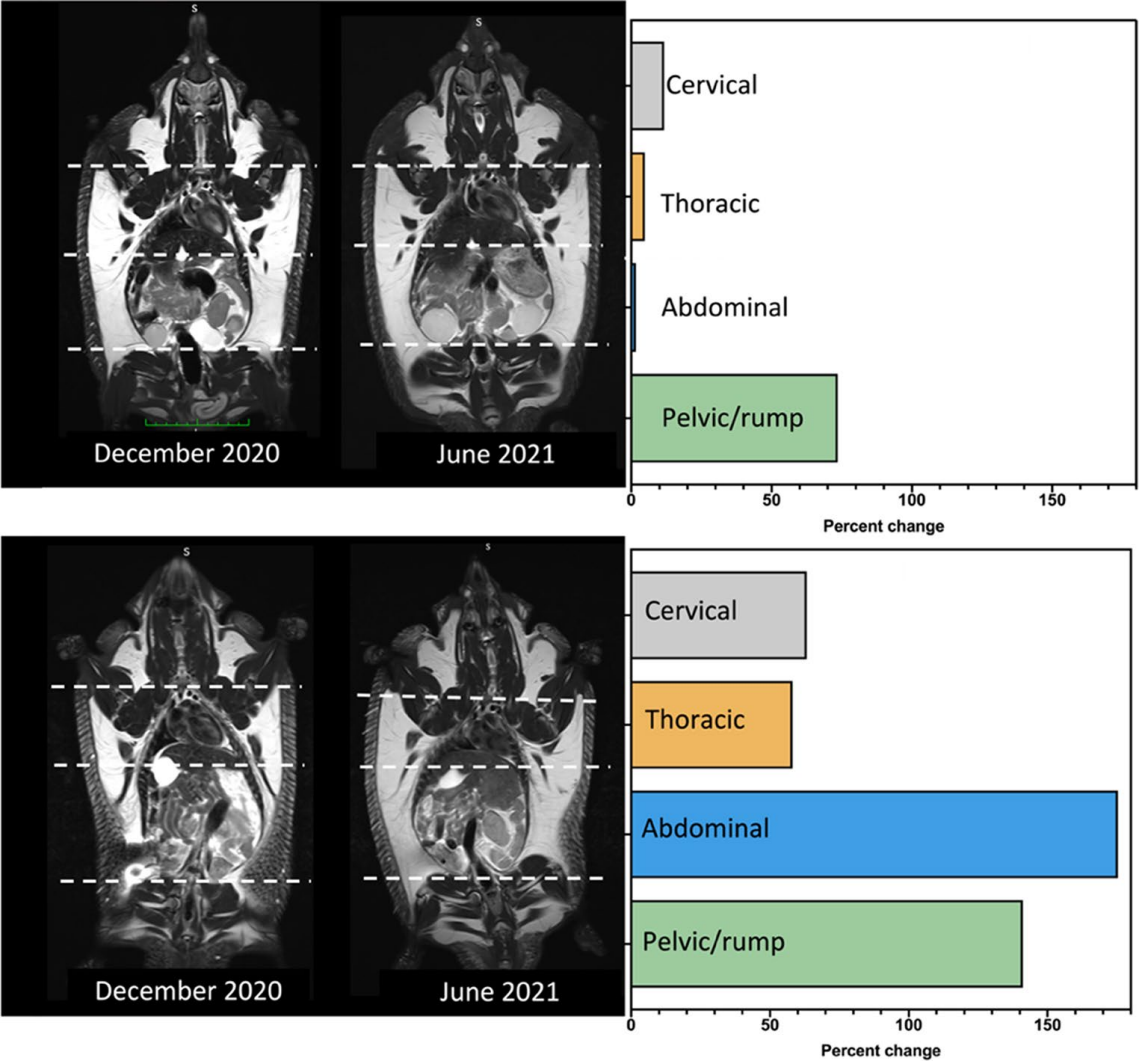
Relative change (%) in adipose tissue between MRI scans conducted two months after the end of (December 2020) and one month before (June 2021) the echidna breeding season (July–October) in a male (**a**) and a female (**b**) echidna

MRI scans clearly revealed the distribution of subcutaneous (SC) adipose tissue in both male and a female echidnas two months after the end of (December) and one month before (June) the breeding season (July to October; Figs. 5 and 6). SC fat was visible in the dorsolateral aspects of both echidnas from the base of the skull to the base of the tail. Adipose tissue in other locations (e.g. intra-abdominal, mesenteric, visceral, gonadal) while visible by MRI, was more difficult to quantify. Use of the segmentation tool (MedSeg) to quantify the volume of SC adipose tissue in the 3 mm coronal slices resulted in very good agreement (*k* = 0.89–0.93; Table 6). The male echidna had adipose tissue in the December and June scans corresponding to 51.2 and 55.6 mL, respectively; equating to an 8.6% relative increase between scans (Fig. 6; Table 6). Additionally, male testes were larger in the June MRI scan (June: 3.0 × 3.2 cm Vs. Dec: 2.0 × 2.5 cm) confirming seasonal testicular recrudescence. The female echidna had adipose tissue in December and June corresponding to 30.0 and 62.1 mL, respectively; equating to a 107.0% relative increase between scans (Fig. 6; Table 6). For the cervical, thoracic, abdominal, and pelvic/rump regions, the relative change in SC fat between scans for the male echidna was 11.7, 4.9, 1.7 and 73.6%, respectively, and 63.2, 58.2, 175.4, and 141.1%, respectively, for the female echidna (Fig. 6; Table 6).

**Table 6:** Adipose tissue (mL) in a male and female echidna as represented by a 3 mm coronal scan two months after the end of (December 2020) and one month before (June 2021) the echidna breeding season (July to October) showing the relative % change between measures and intra-rater reliability scores (*k*) of manual adipose tissue segmentation

Body region	Adipose tissue (mL) and intra-rater reliability of manual adipose tissue segmentation (k)		Relative change in adipose tissue between scans (%)
	December	June	
Male			
Cervical	11.2 (*0.93*)	12.5 (*0.92*)	11.7
Thoracic	16.9 (*0.89*)	17.8 (*0.91*)	4.9
Abdominal	20.5 (*0.88*)	20.8 (*0.91*)	1.7
Pelvic/rump	2.6 (*0.86*)	4.5 (*0.89*)	73.6
Whole body	51.2 (*0.89*)	55.6 (*0.91*)	8.6
Female			
Cervical	8.2 (*0.90*)	13.3 (*0.91*)	63.2
Thoracic	8.3 (*0.89*)	13.1 (*0.90*)	58.2
Abdominal	8.8 (*0.88*)	24.2 (*0.92*)	175.4
Pelvic/rump	4.7 (*0.84*)	11.4 (*0.88*)	141.1
Whole body	30.0 (*0.90*)	62.1 (*0.93*)	107.0

## Discussion

The aim of our longitudinal study was to quantify the relationships between seasonal changes in fat deposition, nutritional supply and circulating leptin concentrations in the short-beaked echidna in addition to visualising fat distribution before and after the breeding season. Specifically, we predicted that circulating leptin levels would be directly proportional to adiposity during most of the year, but that a change in this relationship would occur during the pre-breeding season to allow increased fat. While body fat and plasma leptin concentrations were not correlated in either sex maintained on a standard diet, female echidnas provided with supplemented nutrition (two months before and during the breeding season) demonstrated significantly higher plasma leptin levels during the breeding season. This observation is consistent with the interpretation that this species exhibits leptin resistance. However, additional studies are required to confirm both the robustness of this observation, as well as to determine any underlying mechanism.

The gene expression data confirmed that the expression of the leptin receptor was not a limiting factor for the actions of leptin in the echidnas. The leptin receptor was present in all major organs of the body, including the reproductive organs of both males and females, consistent with leptins actions in eutherians (Pereira et al. 2021; Misch and Puthanveetil 2022). In addition, *LEPTIN* itself was also present in all fat tissues, alongwith *LEPR*, indicating the potential for leptin to regulate its own expression levels. It would be interesting to see if expression levels of the receptor change across the different tissues, between the breeding and non-breeding seasons and between juveniles and adults. However this requires further investigation.

For echidnas maintained on a standard diet, DEXA scans showed that overall, female echidnas have significantly higher body fat % than their male conspecifics (30.4 vs. 23.8%, respectively), but the difference in fat varies between periods; female echidnas have significantly higher fat in the pre-breeding season, but not in the post breeding season (unfortunately there were inadequate data to statistically analyse the breeding season). Sex differences in body fat are common across mammalian species with females often having higher body fat than males (e.g. rhesus macaque, *Macaca mulatta*, Schwartz and Kemnitz 1992; Syrian hamster, *Mesocricetus auratus*, Krol et al. 2005; red backed voles, *Myodes*, Schulte-Hostedde et al. 2001). This might reflect differences in anticipated energy expenditure during the breeding season with females investing substantial energy reserves beyond courtship and copulation to successfully develop, lactate and wean young.

While no correlation of body fat percentage and plasma leptin levels were observed in the echidnas maintained on a standard diet, this may be a consequence of a lack of data or statistical power (with only five body fat data points per echidna). However, manipulation of the diet of five female echidnas before (May and June) and during the breeding season (July–October) did translate to a significant elevation of plasma leptin levels during the breeding season. Whilst more data are needed, our data suggest that leptin resistance may be part of the seasonal regulation of fat deposition in the short-beaked echidna; indeed, the expected effects of leptin (e.g. appetite suppression) were not evident during the supplementary feeding phase, coinciding with a period (breeding season) where fattening would be advantageous.

Interestingly, in females provided with supplemented nutrition, plasma leptin levels were declining or had dropped by the October sampling point, before supplemental feeding had ceased (end of October) suggesting that food supplementation may not be the only factor associated with the elevated plasma leptin levels in reproductive females during the breeding season. Leptin has a role modulating reproduction by ensuring adequate energy balance for the attainment of puberty and the optimal timing of reproduction and influences reproductive function by providing signals to the hypothalamus to modulate GnRH neuronal activity (Hill et al. 2008). A decrease in body and fat mass, concurrent with increased leptin-like immunoreactivity has been observed during egg-laying in the wild female European starlings (*Sturnus vulgaris*), suggestive of a link between egg production and leptin in birds (Kordonowy et al. 2010). Although study animal numbers were too low in our study to confirm a link between leptin and egg production in echidnas, the leptin receptor was present in the ovary of female echidnas and the increased plasma leptin levels detected during the breeding season in supplemental fed reproductive females suggest this is an area that warrants further investigation.

Our captive study sets a baseline for future studies in wild echidna, but quantifying echidna leptin levels is challenging. Despite conducting this study in a controlled environment, we observed plasma leptin levels to be highly variable within and between individual echidnas suggesting any study of wild populations would need multiple sampling events to capture this variation. While we have demonstrated that this variation did not appear to be related to environmental variables or reproductive activity or the echidna’s ability to enter torpor or hibernation (Nicol and Anderson 2007; reviewed in Nicol 2017), the echidna is known to exhibit high levels of heterothermy with body temperature ranging from 28 to 35 °C; maximum temperatures occur during active periods and minimum temperatures occur during rest (Nicol and Anderson 2008; Wallage et al. 2015). We observed two females to have low plasma leptin levels during torpor, and an additional female was in torpor as leptin levels had started to increase. Thus, it is unclear whether variability in body temperature could affect circulating leptin concentrations in captive echidnas in SE Queensland; a phenomenon which could be explored in future studies by implanting echidnas with internal temperature loggers as described by Wallage et al. (2015).

MRI clearly showed SC fat extending dorso-laterally from the base of the skull to the base of the tail in both the male and female echidna that we analysed. In the pre-breeding season, both sexes had considerable fat deposition in the pelvic/rump region, but the female echidna accumulated most fat in the abdominal region; a pattern of fat deposition that appears consistent with that of other seasonally reproducing or hibernating species (Prestrud and Nilssen 1992). Both echidnas participated in reproductive activity between MRI scans (Dutton-Regester et al. 2021, 2022). Despite the female echidna having deposited a three-fold increase of fat in the abdominal region before the breeding season, in the post-breeding season, she appeared to have a hollowing of the waist region, consistent with a loss of body condition that was not evident in the male echidna. Other adipose tissue deposits (e.g. intra-abdominal, mesenteric, visceral, gonadal), while visible using MRI, may be better visualised and/or quantified using dissection of cadavers. Interestingly, LeeHong et al. (2014) found that intra-abdominal fat associated with the gonads and mesentery was only conspicuous in highly conditioned animals. Although further observations are required to confirm the energetic value of the intra-abdominal fat deposits in the echidna, it appears that this species stores the bulk of their adipose tissue in the SC layer, similar to other mammalian species (Pond et al. 1992; Prestrud and Nilssen 1992).

In conclusion, seasonal changes in body mass, body fat percentage and plasma leptin concentrations were not observed in either echidna sex maintained on a standard diet. However, we observed a significant elevation in plasma leptin levels after periodically increasing the diet of five female echidnas, indicating leptin resistance. Given our limited body fat data set and the unique biology of echidnas (i.e. daily heterothermy and torpor) whether leptin functions as an adipostat in the echidna remains inconclusive. In effort to resolve this, future studies should manipulate the diet of echidnas implanted with temperature loggers in addition to collecting more frequent measures of body fat and of plasma to increase statistical power.
